# Reliability of a new measure to assess modern screen time in adults

**DOI:** 10.1186/s12889-019-7745-6

**Published:** 2019-10-28

**Authors:** Maricarmen Vizcaino, Matthew Buman, C. Tyler DesRoches, Christopher Wharton

**Affiliations:** 1 Radical Simplicity Lab, College of Health Solutions, Arizona State University, 550 N 3rd Street, Phoenix, AZ 85004 USA; 2 College of Health Solutions, Arizona State University, Phoenix, USA; 3 School of Sustainability/ School of Historical, Philosophical and Religious Studies, Arizona State University, Tempe, USA

**Keywords:** Screen-time, Television, Smartphone, ICC, SEM, Reliability

## Abstract

**Background:**

Screen time among adults represents a continuing and growing problem in relation to health behaviors and health outcomes. However, no instrument currently exists in the literature that quantifies the use of modern screen-based devices. The primary purpose of this study was to develop and assess the reliability of a new screen time questionnaire, an instrument designed to quantify use of multiple popular screen-based devices among the US population.

**Methods:**

An 18-item screen-time questionnaire was created to quantify use of commonly used screen devices (e.g. television, smartphone, tablet) across different time points during the week (e.g. weekday, weeknight, weekend). Test-retest reliability was assessed through intra-class correlation coefficients (ICCs) and standard error of measurement (SEM). The questionnaire was delivered online using Qualtrics and administered through Amazon Mechanical Turk (MTurk).

**Results:**

Eighty MTurk workers completed full study participation and were included in the final analyses. All items in the screen time questionnaire showed fair to excellent relative reliability (ICCs = 0.50–0.90; all < 0.000), except for the item inquiring about the use of smartphone during an average weekend day (ICC = 0.16, *p* = 0.069). The SEM values were large for all screen types across the different periods under study.

**Conclusions:**

Results from this study suggest this self-administered questionnaire may be used to successfully classify individuals into different categories of screen time use (e.g. high vs. low); however, it is likely that objective measures are needed to increase precision of screen time assessment.

## Background

Screen time among adults represents a continuing and growing problem in relation to health behaviors and health outcomes. Extended periods of screen use – interaction with electronic devices that primarily deliver content via screen-based displays – have been associated with multiple adverse health outcomes including obesity, type 2 diabetes, cardiovascular disease, and early mortality in adults [1–5]. Associations remain even when controlling for age, health history, and health-related behaviors such as smoking, alcohol consumption, physical activity and dietary intake [1–5].

The impact of screen use on health is a complex phenomenon that may go above and beyond the sedentary behavior that may result from prolonged sitting. Television watching has been previously associated with poor dietary choices in part due to heavy commercial advertisement [6]. The frequent use of smartphones has been associated to sleep disturbances [7, 8] potentially resulting from exposure to radio frequency electromagnetic fields that can affect brain physiology [9]. The use of e-readers in the evening has also been shown to affect sleep via suppression of melatonin secretion and alterations to the circadian rhythm [10]. In addition, long periods of screen time have been associated with poor mental health including depression [11, 12], which in turn may disturb the hypothalamic adrenal axis adversely affecting immune function and metabolism [13].

The problem is unlikely to resolve itself given the near ubiquity of screens in modern life. The average American family, for example, owns three television sets [14] and among television-owning households, close to 60% own at least one internet-enabled device such as a Smart TV or a video game console [15]. In addition, approximately 77% of Americans own a smartphone and 78% have at least one desktop or laptop at home [16, 17].

Moreover, with the rapid evolution of screen-based technologies, the landscape of media use has changed at an equally dramatic pace. For example, even though television remains the most widely used screen-based device among US adults, its popularity has steadily decreased in recent years [18]. Simultaneously, the use of portable screen-based devices increased quickly. Recent Nielsen data showed that use of ‘apps’ and internet services on a smartphone more than doubled from 2015 to 2017, and use of apps and web services on tablets increased in the same period by 70% [15].

Moreover, television-connected devices (e.g. gaming consoles, smart TVs) have given rise to the phenomenon of “binge watching” – viewing multiple episodes of a television show in a single sitting – which is observed across multiple generations [19]. Hence, these newer devices represent critical considerations for capturing an updated view of how adults interact and spend time with screens.

Past research on screen time has focused almost exclusively on quantifying “screen use” as the number of hours of television viewing in a given week [20] and only a small number of studies have aggregated television/videos/games and computer use [21, 22]. As such, the instruments available within the health literature designed to capture screen time behavior generally only measure television viewing time and sometimes non-occupational computer use alongside other sedentary behaviors such as reading, driving, and socializing. To the authors’ knowledge, no instrument currently exists that quantifies the use of other specific screen-based devices alongside use of televisions and computers. This represents a critical gap in screen-time assessment as it might be important to distinguish how and when different modern devices are used along with traditional screen-based devices, as well as the variety of ways that different types of screens might be used (e.g. committed use vs. use only as background noise), which may possibly be associated with different patterns of sedentary behavior activity and health outcomes.

Although a variety of screen time tracking applications (‘apps’) and devices are available for smartphones, tablets, PCs, and televisions, to the authors’ knowledge no single application or tool exists that can track all devices at the same time for overall estimation of total screen time. Asking participants to track usage per device via various apps could be burdensome and possibly reveal more information than a participant might want, such as minutes on social media or on particular apps. Also, current television screen time trackers can be significantly expensive and could potentially require research personnel to perform a home visit for installation.

A questionnaire with strong psychometric properties can be a useful research tool that estimates global screen time in a simple, fast, no-cost, and completely anonymous manner. This questionnaire then may be used with online anonymous samples and/or large clinical studies that examine the association between modern screen time and health outcomes. Therefore, the purpose of this study was to develop and assess the reliability of a new screen time questionnaire designed to quantify use of multiple popular screen devices among the US adult population, including television, television-connected devices, laptops, smartphones, and tablets.

## Methods

### Screen time questionnaire

An 18-item screen time online questionnaire was created to quantify use of commonly used screen-based devices (please see Additional file 1: Screen Time Questionnaire). Five different categories of devices were created – TV, TV-connected devices (e.g. streaming devices, video game consoles), laptop/computer, smartphone, and tablet – based on the classification scheme used by publicly available reports on screen time usage among the American population [15, 23–25]. These categories were believed to appropriately reflect the purpose of the questionnaire, which is to quantify different forms of screen time use among American adults. For instance, it was important to differentiate between TV and TV-connected devices because of the growing trend of adults watching subscription-based video and on-demand content instead of regularly programmed television [26, 27].

Moreover, it was considered appropriate to include a variety of internet-enabled devices, such as game consoles and multimedia devices, under the same category (TV-connected devices) since the use of these devices would reflect the same sedentary behavior of sitting for prolonged periods of time that is initiated at any time during the day, instead of being a behavior that is driven by a predetermined schedule like TV programming.

In the present online questionnaire, participants were instructed to estimate total time spent in hours and minutes using each device. Total time for each screen-based device was quantified in minutes (e.g. 1 h and 30 min = 90 min). Because screen time use shows variation throughout the day and week [24], the questionnaire further inquired about screen use during an average weekday, an average weeknight, and an average weekend day (Saturday or Sunday) separately.

In addition, because the use of a screen while performing other activities that require body movement would not represent sedentary behavior that impacts physical health and thus health outcomes, the questionnaire was divided into sections exploring screen use as primary activity and screen use in the background. Taking into account the time spent only in a primary activity has been used previously by time-use surveys [28]. The “primary activity” was defined in the survey as “the main activity you are engaged in rather than using a television or other screen in the background while performing another activity, such as cooking or exercising.” Background use was defined as “the use of a television or another screen near you while performing other activities such as exercising, cooking, and interacting with family/friends.”

Lastly, basic demographic information was also collected (e.g., age, sex) along with self-reported height, weight, and physical activity levels. Body mass index (BMI) was calculated by dividing weight in kilograms over height in meters squared. Physical activity levels were estimated with the Stanford Leisure-Time Activity Categorical Item (L-Cat), which has previously shown adequate psychometric properties [29].

### Data collection

The questionnaire was delivered online using Qualtrics and administered through Amazon Mechanical Turk (MTurk). U.S. workers on MTurk are more similar to the U.S. population compared to subjects recruited from traditional university subject pools and provide greater diversity in terms of age, ethnicity, and socio-economic status [30–32]. In addition, previous studies have shown that MTurk samples can provide valid and reliable data for health and social science research [30–32]. For instance, Casler, Bickel, & Hackett adapted a behavioral, face-to-face task to an online test and found that test results were almost identical between a standard sample of college students attending an in-person lab session and participants recruited online through MTurk [33].

Data collection was conducted between March and October 2018. To achieve a high level of data quality, multiple strategies were employed such as attention checks and a ‘captcha’ verification to exclude spam and automated responses. Inclusion criteria included adults 18 years of age and older who watched television or a television-connected device for at least 2 h daily and owned at least one other screen-based device other than a television, English speakers, and current residents of the US. The study was approved by the Institutional Review Board of a university in the Southwest of the United States.

Eligible MTurk workers signed an electronic informed consent form and were redirected to the Qualtrics survey where they were asked about their demographics, height, weight, current physical activity levels, and screen time (time 1). At the end of the survey, a completion code and a new Qualtrics link was provided. Participants were instructed to access the link 3 days later to complete the screen-time questionnaire a second time (time 2). Afterwards, they were provided a final code to enter into the MTurk website in order to receive a $5 payment. The research team included only participants whose data entries were separated by at least 3 days.

### Data analyses

Data were analyzed using the Statistical Package for the Social Sciences (SPSS) version 21.0. Because all variables under investigation were found positively skewed violating normality assumptions, even after transformation attempts, screen time variables are presented as medians and interquartile ranges. Test-retest reliability of the screen-time questionnaire was assessed via two different approaches. Relative reliability was assessed through intra-class correlation coefficients (ICCs) using a two-way mixed effects, single measurement, absolute agreement model. Values represent the following: less than 0.40 - poor reliability, between 0.40 and 0.59 – fair reliability, between 0.60 and 0.74 - good reliability, and greater than 0.75 - excellent reliability [34]. Relative reliability refers to consistency of the position of individuals in the group relative to others, and hence allows for the determination of how well participants can be distinguished from each other regardless of measurement errors [34, 35].

Absolute reliability, or agreement, was assessed through the standard error of measurement (SEM) using the following formula:

$$ \mathrm{SEM}={{\sqrt{\sigma}}_{\mathrm{e}}}^2 $$

where *σ*_e_^2^ is the error variance in a repeated measures analysis of variance (ANOVA). In addition, two-sided 95% confidence intervals were estimated from the following formula:

$$ \surd \left[\mathrm{SSE}/{x^2}_{\mathrm{a},\mathrm{dfe}};\mathrm{SSE}/{x^2}_{1-\mathrm{a},\mathrm{dfe}}\right] $$

where SSE is the sum of squares error from the repeated measures ANOVA, *x*^2^_a, dfe_ is the chi-square value for the probability level alpha and dfe is the degrees of freedom associated with SSE. Absolute reliability or agreement refers to the consistency of scores of individuals, and therefore indicates how similar the scores for repeated measures are when measurement error (systematic and random) is present [34, 35].

Sample size calculation was performed using the Donner & Eliasziw approach [36] using an alpha level of 0.05 and a power of 0.80. For an ICC between 0.4 and 06, corresponding to acceptable reliability, a sample size of at least 86 is required. On the other hand, for an ICC between 0.6 and 0.8, corresponding to good to excellent reliability, a sample size of at least 39 is required [36]. In addition, it was determined that two observations were adequate for the present analysis. Previous authors have suggested that when examining the reproducibility of a questionnaire, two observations per subject are more adequate in order to avoid memory effect; also, the width of the 95 confidence interval does not change between two and three observations [37]. Due to follow-up issues with previous data collection using MTurk by our research team, it was decided to request the participation of 200 workers in order to approximate a sample size of 86.

## Results

A total of 170 MTurk workers enrolled in the study; 83 participants did not take the second survey and seven participants were excluded for varied reasons (e.g. failing the check question, incomplete data, etc.). Hence, a total of 80 participants completed full study participation and were included in the final analyses. The majority of participants were male (62.5%), 35 years of age or younger (60%), non-Hispanic White (91.3%), single (58.8%), employed full-time (78.8%), had a Bachelor’s or higher degree (58.8%), had a household income of less than $60,000 per year (68.8%), and had a BMI of 26.7 or less (58.8%). Additionally, approximately half (53.8%) reported engaging in current physical activity recommendations of at least 30 min or more of moderate-intensity activity 5 days per week [38].

Minutes spent on each screen device as a primary form of activity are presented in Table 1. The most commonly used screen during a weekday was a laptop/computer, followed by television and television-connected devices, smartphone, and tablet. The pattern was similar for screen use during a weeknight and a weekend day. Lastly, participants reported the most background screen use during the weekend with an aggregate of approximately 3 h across devices, whereas background screen use during an average weekday and average weeknight was approximately 2 h (see Table 2).

**Table 1:** Minutes spent on different screen devices as a primary activity during an average weekday, weeknight, and weekend day by MTurk participants (*N* = 80)

		Weekday		Weeknight		Weekend	
		*Median*	*IQR*	*Median*	*IQR*	*Median*	*IQR*
TV	Time 1	120	60–180	60	30–120	120	60–240
	Time 2	120	60–180	60	60–120	120	60–240
TV-connected devices	Time 1	120	60–180	60	30–120	120	60–202.5
	Time 2	120	60–131.3	90	30–120	120	60–240
Laptop/ computer	Time 1	420	245–600	120	60–180	240	120–360
	Time 2	405	242.5–600	120	60–180	255	120–360
Smartphone	Time 1	60	60–120	60	30–60	90	60–120
	Time 2	60	60–120	60	30–120	60	48.8–120
Tablet	Time 1	15	0–60	0	0–30	0	0–60
	Time 2	0	0–60	0	0–60	0	0–60

*TV* Television, *IQR* Interquartile range

**Table 2:** Minutes exposed to background screen use during an average weekday, weeknight, and weekend by MTurk participants (*N* = 80)

	Weekday		Weeknight		Weekend	
	*Median*	*IQR*	*Median*	*IQR*	*Median*	*IQR*
Time 1	120	60–262.5	120	60–180	180	120–360
Time 2	120	75–285	120	60–202.5	165	63.8–360

*IQR* Interquartile range

Relative reliability results for the screen-time questionnaire are presented in Table 3. Items inquiring about television, laptop/computer, smartphone, and tablet use during a weekday and the three questions associated with screen use in the background showed good to excellent reliability (ICCs = 0.61–0.90). Items inquiring about screen use during a weeknight showed fair to excellent reliability (ICCs = 0.50–0.82). Items inquiring about screen use during a weekend day showed excellent reliability (ICCs = 0.84–0.87), except for smartphone use (ICC = 0.16).

**Table 3:** Intra-class correlation results for the different sections of the screen-time questionnaire

		ICC	95% CI	*p* value
Weekday	Television	0.79	0.69–0.86	0.000
	Television-connected devices	0.90	0.85–0.94	0.000
	Laptop/computer	0.89	0.83–0.92	0.000
	Smartphone	0.85	0.78–0.90	0.000
	Tablet	0.61	0.45–0.73	0.000
Weeknight	Television	0.50	0.31–0.64	0.000
	Television-connected devices	0.82	0.74–0.88	0.000
	Laptop/computer	0.68	0.55–0.78	0.000
	Smartphone	0.66	0.51–0.77	0.000
	Tablet	0.68	0.54–0.78	0.000
Weekend day	Television	0.87	0.80–0.91	0.000
	Television-connected devices	0.84	0.76–0.89	0.000
	Laptop/computer	0.84	0.76–0.89	0.000
	Smartphone	0.16	−0.05 – 0.37	0.069
	Tablet	0.74	0.62–0.82	0.000
Background use	Weekday	0.86	0.79–0.91	0.000
	Weeknight	0.79	0.69–0.86	0.000
	Weekend day	0.73	0.61–0.82	0.000

*ICC* Intra-class correlation coefficient, *CI* Confidence interval

Absolute reliability results for the screen-time questionnaire is presented in Table 4. Overall, SEM values were large for all types of screens across the different periods under study. However, measurement error was smaller among the items inquiring about television, laptop/computer, smartphone, and tablet use during an average weeknight, as were confidence intervals. Among the different types of screen, television-connected devices and laptop/computer use during a weekday and weeknight showed the most precision, whereas smartphone use during a weekend day showed the greatest measurement error.

**Table 4:** Standard error of measurement results for the different sections of the screen-time questionnaire

		SEM (min)	95% CI
Weekday	Television	55.10	46.53–63.55
	Television-connected devices	33.42	28.22–38.54
	Laptop/computer	99.81	84.27–115.10
	Smartphone	27.80	23.47–32.06
	Tablet	38.27	32.31–44.13
Weeknight	Television	45.82	38.68–52.84
	Television-connected devices	36.93	31.18–42.59
	Laptop/computer	78.07	65.91–90.03
	Smartphone	30.01	25.34–34.61
	Tablet	21.17	17.87–24.41
Weekend day	Television	47.70	40.27–55.01
	Television-connected devices	51.35	43.36–59.22
	Laptop/computer	81.39	68.26–93.24
	Smartphone	146.89	124.01–169.39
	Tablet	24.66	20.82–28.44
Background use	Weekday	93.07	78.58–107.33
	Weeknight	56.12	47.38–64.72
	Weekend day	129.73	109.53–149.60

*SEM* Standard error of measurement, *CI* Confidence interval

## Discussion

The present study assessed the reliability, both relative and absolute, of a newly developed screen time questionnaire. The questionnaire quantifies the use of a variety of screen-based devices that better reflects the more varied screen-time behaviors of US adults, and it makes the distinction between different contexts in which screens may be used (actual use vs. background use as well as day vs. evening vs. weekend use). To our knowledge, this is the first questionnaire that inquiries about the use of commonly used screens beyond television and computers among the US adult population.

Current questionnaires predominantly focus on overall sedentary behavior and estimate “screen time” by summing hours spent per day or week watching television/television or computer/television or playing games [39], but they do not quantify other forms of screen use such as tablets and smartphones. The questionnaire by Marshall et al. [40] additionally included an item asking respondents to report hours spent sitting during leisure time engaged in other activities not including television, such as visiting friends, watching movies away from the home, or dining. The inclusion of this catch-all “other” section might potentially include the use of additional screen devices; however, the questionnaire does not include a way to explicitly make this distinction.

The present questionnaire is also the first one to include a separate assessment for weeknights and background use. It has been previously reported that a large percentage of Americans engage in screen time during the evenings compared to the rest of the day [24], and hence screen time specifically during weeknights may serve as a valuable target of change for interventions that aim to reduce overall sedentary behavior. Similarly, prior research has found that background television use has detrimental effects on cognitive processing [41, 42] and thus background screen time may be an important target of change for behavioral interventions that seek to improve overall well-being including intellectual development and learning.

Results from this study compare favorably to previous test-retest reliability studies in a number of ways. Previous questionnaires have shown ICCs ranging from 0.62 to 0.69 for computer use [39], whereas the present questionnaire demonstrated higher ICCs ranging from 0.68 to 0.89 across the different periods under study, indicating good to excellent relative reliability. Prior questionnaires have also found ICCs ranging from 0.32 to 0.82 for television viewing [39, 43], whereas this questionnaire found slightly higher ICCs ranging from 0.50 to 0.87. In addition, items inquiring about additional types of screen such as television-connected devices and tablets showed ICCs ranging from 0.61 to 0.90, indicating good to excellent relative reliability, except for smartphone during an average weekend day. As a result, the present questionnaire was able to successfully distinguish between low- and high- users of a variety of different screen-based devices in addition to television and computer.

Furthermore, to our knowledge this is the first study that examines the absolute reliability of a self-administered questionnaire inquiring about screen time in adults. Our analyses indicated that even though our questionnaire would be able to adequately classify individuals into different categories (e.g. heavy screen time users vs. moderate screen time users) as evidenced by high ICCs, this self-report measure may not provide consistent results over repeated assessments as indicated by measurement error. The results indicated that the most precise estimation of screen time was for television-connected devices and laptop/computer use during a weekday and weeknight, whereas the least precise was smartphone use during the weekend. This may the result of distinct periods of time when people watch favorite TV shows and/or engage in specific computer tasks on a daily basis allowing for easy recall and thus more precise estimation of screen time. On the other hand, during weekends when individuals have more free time at their disposal, it could be difficult to precisely estimate how much screen time is spent with a particular device, particularly smartphones that provide easy access to browsing at any time during the day and at almost any location.

These results highlight the importance of using objective measures in addition to self-administered questionnaires when assessing screen time. Objective measures such as television timers and smartphones apps may provide a more accurate quantification of time spent on a variety of screen devices, which may be particularly helpful for studies that seek to demonstrate a reduction in screen time after a behavioral intervention that is due to real change and not simply due to measurement error.

Lastly, these results emphasize the need to quantify different types of screen use in order to provide a more accurate representation of overall screen time for adults. Participants in this study reported spending more hours combined using television-connected devices and smartphones compared to television alone across all periods under study; as such, including television only as a measure of “screen time” may substantially underestimate true screen time and potentially sedentary behavior that may contribute to obesity and other chronic conditions.

### Future directions

Some limitations to this study exist, including a brief test-retest reliability period. Future studies should examine the stability of reliability coefficients using a longer test-retest period. Also, the questionnaire needs to be examined for additional psychometric properties such as convergent and discriminant validity. Future studies could employ measures to objectively quantify hours of screen use (e.g. television monitors, smartphone apps) in addition to activity monitors that estimate sedentary behavior (e.g. activPAL™) and compare these against self-reported screen time as measured by the present screen-time questionnaire.

Lastly, it would be interesting to develop another questionnaire that quantifies the concurrent use of different screen-based devices. A recent meta-analysis found that media multitasking has a detrimental impact on cognitive outcomes such as attention, comprehension, and recall; nevertheless, little is known about the consequences of media multitasking on long-term mental health [44].

## Conclusions

To the authors’ knowledge, this is the first study to test reliability (both relative and absolute) of a screen-time questionnaire tool that incorporates a greater variety of modern screen-based devices. Relative reliability results suggest this tool could be used to appropriately classify individuals into different screen time categories across multiple devices (e.g. heavy users of television vs. light users of television; heavy users of laptop/computer vs. moderate users of laptop/computer). Our results also emphasize the importance of assessing diverse types of screen to obtain an accurate representation of overall screen time.

## Supplementary information

**Additional file 1.** Screen-time Questionnaire. The questionnaire includes all items used to quantify the use of a variety of modern screen-based devices.

## Data Availability

The datasets used and/or analyzed during the current study are available from the corresponding author on reasonable request.

## Abbreviations

ANOVA: Analysis of variance
BMI: Body Mass Index
ICCs: Intra-class correlation coefficients
L-Cat: Stanford Leisure-Time Activity Categorical Item
MTurk: Amazon Mechanical Turk
SEM: Standard error of measurement
SPSS: Statistical Package for the Social Sciences
