# Supplementary material for: Reliability of a new measure to assess modern screen time in adults
Source: BMC Public Health. 2019 Oct 28;19:1386. doi: 10.1186/s12889-019-7745-6 (PMC6816215; doi:10.1186/s12889-019-7745-6)
Supplement: Supplementary file 1 — Additional file 1. Screen-time Questionnaire. The questionnaire includes all items used to quantify the use of a variety of modern screen-based devices. [file 12889_2019_7745_MOESM1_ESM.docx]

Screen-time Questionnaire

For the following set of questions, ***primary activity*** is defined as the main activity you are engaged in rather than using a television/other screen in the background while performing another activity such as cooking or exercising.

| **Screen use on an average weekday**  Thinking of an average weekday (from when you wake up until you go to sleep), how much time do you spend using each of the following types of screen as the primary activity?     You must answer both hours and minutes. **If zero please type "0" in the box.** | | |
| --- | --- | --- |
|  | Hours | Minutes |
| Television |  |  |
| TV-connected devices (e.g. streaming devices, video game consoles) |  |  |
| Laptop/computer |  |  |
| Smartphone |  |  |
| Tablet |  |  |

| **Screen use on an average weeknight**  Now, thinking of an average weeknight (from when you return from work until you go to sleep), how much time do you spend using each of the following types of screen as the primary activity?  You must answer both hours and minutes. **If zero please type "0" in the box.** | | |
| --- | --- | --- |
|  | Hours | Minutes |
| Television |  |  |
| TV-connected devices (e.g. streaming devices, video game consoles) |  |  |
| Laptop/computer |  |  |
| Smartphone |  |  |
| Tablet |  |  |

| **Screen use on an average weekend day**  Now, thinking of an average weekend day (Saturday or Sunday), how many hours over the course of the whole day (from when you wake up until you go to sleep) do you spend using each of the following types of screen as the primary activity?  You must answer both hours and minutes. **If zero please type "0" in the box**. | | |
| --- | --- | --- |
|  | Hours | Minutes |
| Television |  |  |
| TV-connected devices (e.g. streaming devices, video game consoles) |  |  |
| Laptop/computer |  |  |
| Smartphone |  |  |
| Tablet |  |  |

For the following set of questions, **background screen** is defined as the use of a television or another screen near you while performing other activities such as exercising, cooking, and interacting with family/friends.

Thinking about a regular weekday (Monday through Friday), on average, how many hours **over the course of the whole day** (from when you wake up until you go to sleep) are you exposed to background screen use?

 *Example: If you exercise in the morning for one hour while watching the TV news, you use your smartphone for one hour while eating lunch and an additional 30 minutes while eating dinner, you would estimate that you are exposed to 2 hours and 30 minutes of background screen use per day.*

|  | Hours | Minutes |
| --- | --- | --- |
| Background screen use on a regular weekday |  |  |

Now we want to ask about background screen use **during the evening specifically**. On average, how many hours per evening (Monday through Friday) are you exposed to background screen use from when you return from work until you go to sleep?   
 
*Example: If you regularly prepare dinner with the television on for one hour, and you keep the television on for an additional hour while using your smartphone for social media use, you can estimate that you are exposed to 2 hours of background screen use every evening.*

|  | Hours | Minutes |
| --- | --- | --- |
| Background screen use on a regular weeknight |  |  |

Now we want to ask about background screen use **during the weekend**. Thinking about a regular weekend day (Saturday or Sunday), on average, how many hours over the course of the whole day (from when you wake up until you go to sleep) are you exposed to background screen use?   
 
*Example: If you have the television on while you do some online shopping for two hours, and you keep the television on when friends come over to visit for an additional two hours, you can estimate that you are exposed to 4 hours of background screen use every evening.*

|  | Hours | Minutes |
| --- | --- | --- |
| Background screen use on a regular weekend day |  |  |
